# Efficacy and safety of a novel dosing strategy for ruxolitinib in the treatment of patients with myelofibrosis and anemia: the REALISE phase 2 study

**DOI:** 10.1038/s41375-021-01261-x

**Published:** 2021-05-20

**Authors:** Francisco Cervantes, David M. Ross, Atanas Radinoff, Francesca Palandri, Alexandr Myasnikov, Alessandro M. Vannucchi, Pierre Zachee, Heinz Gisslinger, Norio Komatsu, Lynda Foltz, Francesco Mannelli, Francesco Passamonti, Geralyn Gilotti, Islam Sadek, Ranjan Tiwari, Evren Zor, Haifa Kathrin Al-Ali

**Affiliations:** 1 Hospital Clínic, IDIBAPS, University of Barcelona, Barcelona, Spain; 2 Royal Adelaide Hospital and Flinders Medical Centre, Adelaide, SA Australia; 3 University Hospital Sveti Ivan Rislki, Sofia, Bulgaria; 4 IRCCS Azienda Ospedaliero-Universitaria di Bologna, Institute of Hematology “Seràgnoli”, Bologna, Italy; 5 Department of Hematology, V.A. Baranov Republican Hospital, Petrozavodsk, Russian Federation; 6 CRIMM, Center for Research and Innovation of Myeloproliferative Neoplasms, AOU Careggi, Department of Experimental and Clinical Medicine, University of Florence, Florence, Italy; 7 Hematology Service, ZNA Stuivenberg, Antwerp, Belgium; 8 Department of Internal Medicine I, Division of Hematology and Hemostaseology, Comprehensive Cancer Center, Medical University of Vienna, Vienna, Austria; 9 Juntendo University School of Medicine, Tokyo, Japan; 10 St Paul’s Hospital, University of British Columbia, Vancouver, BC Canada; 11 University of Insubria, Varese, Italy; 12 Novartis Pharma, East Hanover, NJ USA; 13 Novartis Healthcare Pvt. Ltd, Hyderabad, India; 14 Novartis Pharma AG, Basel, Switzerland; 15 University Hospital Halle, Halle (Saale), Germany

**Keywords:** Chemotherapy, Myeloproliferative disease

## Abstract

Anemia is a frequent manifestation of myelofibrosis (MF) and there is an unmet need for effective treatments in anemic MF patients. The REALISE phase 2 study (NCT02966353) evaluated the efficacy and safety of a novel ruxolitinib dosing strategy with a reduced starting dose with delayed up-titration in anemic MF patients. Fifty-one patients with primary MF (66.7%), post-essential thrombocythemia MF (21.6%), or post-polycythemia vera MF (11.8%) with palpable splenomegaly and hemoglobin <10 g/dl were included. Median age was 67 (45–88) years, 41.2% were female, and 18% were transfusion-dependent. Patients received 10 mg ruxolitinib b.i.d. for the first 12 weeks, then up-titrations of up to 25 mg b.i.d. were permitted, based on efficacy and platelet counts. Overall, 70% of patients achieved a ≥50% reduction in palpable spleen length at any time during the study. The most frequent adverse events leading to dose interruption/adjustment were thrombocytopenia (17.6%) and anemia (11.8%). Patients who had a dose increase had greater spleen size and higher white blood cell counts at baseline. Median hemoglobin levels remained stable and transfusion requirements did not increase compared with baseline. These results reinforce the notion that it is unnecessary to delay or withhold ruxolitinib because of co-existent or treatment-emergent anemia.

## Introduction

Myelofibrosis (MF) is a hematologic neoplasm characterized by dysregulation of the Janus kinase (JAK)/signal transducer and activator of transcription (STAT) signaling pathway involved in normal hematopoiesis, cell growth, and immune function [1–4]. The main features of MF include atypical megakaryocyte morphology, bone marrow fibrosis, anemia, splenomegaly, fatigue, weight loss, night sweats, and risk of progression to acute myeloid leukemia [5, 6]. The only potentially curative treatment for MF, allogeneic stem cell transplantation, can, in practice, only be applied to a minority of patients, due to age constraints and its associated high rate of mortality [7, 8]. Other available treatments in MF are aimed at palliation of symptoms, including splenomegaly, anemia, constitutional symptoms, bone pain, and symptomatic extramedullary hematopoiesis [9, 10].

Most patients with MF present with mild-to-moderate anemia, which worsens as the disease progresses. The anemia of MF is, in part, due to reduced erythropoiesis, but can also be compounded by hypersplenism, the effects of inflammatory cytokines [11], and concurrent causes such as iron, folate or vitamin B12 deficiency, gastrointestinal bleeding or, more rarely, immune hemolysis [12]. Anemia is a criterion used in the diagnosis of primary MF (PMF), post-polycythemia vera MF (PPV-MF), and post-essential thrombocythemia MF (PET-MF) [1, 13, 14], and is an important factor in risk assessment, conferring an adverse prognostic impact [9, 15–17]. Current treatments for anemia in MF include androgens, erythropoietin, immunomodulatory agents (thalidomide, lenalidomide), and prednisone [9]. However, these drugs may not be effective in all patients, and can be associated with side effects that can lead to discontinuation. For example, erythropoietin rarely works in transfusion-dependent (TD) patients, representing about 25% of those with MF [18, 19]. For this reason, new approaches to the management of anemic patients with MF are needed. In this sense, multiple investigational therapies are being explored for the anemia of myelofibrosis, including luspatercept [20] and momelotinib [21].

Ruxolitinib is a potent JAK1/JAK2 inhibitor approved for the treatment of MF-related splenomegaly or symptoms in adults with PMF, PPV-MF, or PET-MF [22]. The recommended starting dose is 5, 15, or 20 mg twice daily (b.i.d.), depending on platelet counts (≥50 to <100 × 10^9^/l, 100 to 200 × 10^9^/l, >200 × 10^9^/l, respectively) regardless of hemoglobin (Hb) level at baseline [22, 23]. Dose-dependent anemia has been observed with ruxolitinib treatment, with Hb levels reaching a nadir between 8 and 12 weeks after commencing treatment and returning to baseline by week 24 [23, 24]. This anemia is usually managed with dose reductions and/or blood transfusion and, unlike the anemia of MF, anemia while on ruxolitinib treatment does not adversely impact overall survival [23–25]. Clinical experience in the use of ruxolitinib in MF has led to the suggestion that the use of a lower starting dose of 10 mg b.i.d. with up-titration could reduce the impact of treatment-related anemia while maintaining therapeutic response in patients who are already experiencing anemia [18, 19]. This approach assumes that starting at a lower dose may alter the rate of the initial Hb decline and the nadir, by decreasing the level of JAK-mediated inhibition of hematopoiesis.

To test the value of this proposed alternative dosing practice, we conducted the REALISE open-label, single-arm, phase 2 study that evaluated the efficacy and safety of a novel dosing strategy of ruxolitinib, consisting of a reduced starting dose (10 mg b.i.d.) with delayed up-titration in patients with MF and anemia (Hb <10 g/dl) [26].

## Materials and methods

The REALISE study was a phase 2, open-label, single-arm study conducted in 20 centers in Europe, Asia, and North America to evaluate the efficacy and safety of an alternative dosing strategy for ruxolitinib in the treatment of anemic MF patients. The protocol was approved by the appropriate Independent Review Board/Independent Ethics Committee/Research Ethics Board at each study location and all patients provided written informed consent.

### Patients

Patients were eligible for enrollment if they had PMF, PET- MF, or PPV-MF; age ≥18 years; palpable spleen (≥5 cm below the left costal margin, measured using a soft ruler during quiet respiration); Hb level <10 g/dl; Eastern Cooperative Oncology Group (ECOG) performance status of 0, 1, or 2; and peripheral blood blasts <10%. PMF was diagnosed according to the 2016 revised International Standard Criteria [1], and PPV-MF or PET-MF according to standard criteria [14], irrespective of JAK2 mutation status. Prior to study entry, coexistent causes of anemia, such as iron, folate or vitamin B12 deficiency, and gastrointestinal bleeding due to hypertensive gastropathy of portal hypertension were excluded. Patients with a history of red cell transfusions were required to have a documented transfusion record for the 12 weeks prior to baseline. Transfusion dependence was defined according to the International Working Group for Myeloproliferative Neoplasms Research and Treatment (IWG MRT) criteria as 6 or more transfusions in the 12 weeks prior to baseline [27].

Exclusion criteria included: prior treatment with any JAK1 or JAK2 inhibitor; inadequate bone marrow reserve at baseline visit, as demonstrated by at least 1 of the following: absolute neutrophil count (ANC) ≤1 × 10^9^/l, platelet count <50 × 10^9^/l, without the assistance of growth factors, thrombopoietic factors or platelet transfusions, and Hb ≤6.5 g/dl despite transfusions; severely impaired renal function (defined by creatinine clearance less than 30 ml/min); inadequate liver function (total bilirubin ≥2.5 × upper limit of normal [ULN] and subsequent determination of direct bilirubin ≥2.5 × ULN or alanine aminotransferase >2.5 × ULN or aspartate aminotransferase >2.5 × ULN); concurrent treatment with a potent systemic inhibitor or inducer of CYP3A4 at the time of screening; acute viral hepatitis or active chronic hepatitis B or C infection or a history of progressive multifocal leukoencephalopathy. Patients with a history of malignancy in the past 3 years, except for treated early stage squamous or basal cell carcinoma, were also excluded.

### Treatment

**Fig. 1:**
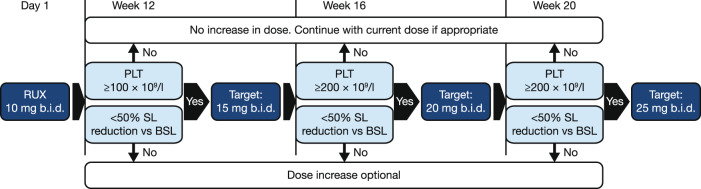
Diagram of up-titration procedure in the REALISE phase 2 study. b.i.d. twice daily, BSL baseline spleen length, PLT platelets, RUX ruxolitinib, SL spleen length.

Eligible patients received a 10 mg oral dose of ruxolitinib b.i.d. for the first 12 weeks of treatment, regardless of their platelet counts at baseline, after which up-titrations of up to 25 mg b.i.d. were permitted, based on platelet counts and efficacy (Fig. 1). Patients with an ANC >0.5 × 10^9^/l and Hb ≥6.5 g/dl were eligible for dose increases. A dose of 15 mg b.i.d. from 12 weeks was targeted for patients with a platelet count ≥100 × 10^9^/l to ≤200 × 10^9^/l; a dose of 20 mg b.i.d. from 16 weeks for patients with a platelet count ≥200 × 10^9^/l; from week 20, 25 mg b.i.d. doses were permitted in patients with a platelet count ≥200 × 10^9^/l who did not achieve a 50% reduction in palpable spleen length (SL). Dose increases were optional for those patients who achieved a ≥50% reduction in SL from baseline. Patients received the study treatment for as long as it was beneficial, up to 48 weeks after the last patient’s first treatment.

### Endpoints and assessments

The primary endpoint was the proportion of patients achieving ≥50% reduction in SL at week 24. Secondary endpoints included transfusion requirements, safety (adverse events [AEs]), changes in Hb, and patient-reported outcomes. Efficacy assessments were: SL, transfusions over time, and patient-reported outcomes as assessed by the 7-point MF score (MF-7), the MF Symptom Assessment Form (MFSAF) version 2.0, and the Patient Global Impression of Change (PGIC). Endpoints were assessed by transfusion-dependence status at baseline, as previously defined [27].

Safety assessments included AEs [28], blood hematologic values, and blood biochemical parameters.

### Statistical analyses

The full analysis set and the safety set were the same. Categorical data are presented as frequencies and percentages. For continuous data, mean, standard deviation, median, 25th and 75th percentiles, minimum, and maximum are used.

The assessment of primary efficacy of study treatment was based on the calculation of the observed proportion of patients with SL response at week 24 and its posterior distribution using a beta-binomial model. Spleen response rate at week 48 was evaluated as the point estimate of the proportion of patients achieving ≥50% reduction in palpable SL at week 48 along with the corresponding exact 95% confidence interval (CI) using the Clopper and Pearson exact method.

## Results

### Patients

A total of 51 patients were treated in the study, of whom 50 had completed or discontinued treatment prior to the data cut-off date of February 16, 2019. The median patient age was 67 (range 45–88) years, and approximately two-thirds of patients had PMF (Table 1; Supplementary Table S1). Most patients had Dynamic International Prognostic Scoring System (DIPSS) intermediate-1 or intermediate-2 disease (9 [17.6%] and 28 [54.9%], respectively), and 10 (19.6%) were classified as DIPSS high-risk. At baseline, 9 patients were TD and 41 were not TD (non-TD). Eleven patients had platelet counts <100 × 10^9^/l at baseline.

**Table 1:** Patient characteristics at baseline^a^.

Characteristics	All patients (*N* = 51)
Age, median (range), years	67 (45–88)
Female, *n* (%)	21 (41.2)
Race, *n* (%)	
White	48 (94.1)
Asian	3 (5.9)
ECOG performance status, *n* (%)	
0	19 (37.3)
1	28 (54.9)
2	4 (7.8)
Type of MF, *n* (%)	
PMF	34 (66.7)
PPV-MF	6 (11.8)
PET-MF	11 (21.6)
Mutational status, *n* (%)	
*JAK2* positive	29 (56.9)
*CALR* positive	7 (13.7)
*MPL* positive	5 (9.8)
Two mutations^b^	3 (5.9)
Triple negative^c^	7 (13.7)
Time since initial diagnosis, median (range), months	14.9 (0.3–222.0)
Prior therapy received, *n* (%)	28 (54.9)
Radiotherapy	1 (2.0)
Danazol	3 (5.9)
Prednisone	2 (3.9)
Erythropoietin	2 (3.9)
Constitutional symptoms present, *n* (%)^d^	29 (56.9)
Palpable SL, median (range), cm	12 (5–35)
DIPSS category, *n* (%)	
Intermediate-1	9 (17.6)
Intermediate-2	28 (54.9)
High	10 (19.6)
Unknown	4 (7.8)
Hb level, median (range), g/dl	8.9 (6.6–11.5^e^)
Platelet count, median (range), ×10^9^/l	181 (55–762)
Platelets <100 × 10^9^/l, *n* (%)	11 (34.5)
WBC, median (range), ×10^9^/l	9.9 (2.7–71.0)
TD^f^, *n* (%)	9 (18.0); *n* = 50

*CALR* calreticulin, *DIPSS* Dynamic International Prognostic Scoring System, *ECOG* Eastern Cooperative Oncology Group, *Hb* hemoglobin, *IWG-MRT* International Working Group for Myeloproliferative Neoplasms Research and Treatment, *JAK2* Janus kinase 2, *MF* myelofibrosis, *MPL* myeloproliferative leukemia protein, *PET* post-essential thrombocythemia, *PMF* primary myelofibrosis, *PPV* post-polycythemia vera, *SL* spleen length, *TD* transfusion-dependent, *WBC* white blood count.

^a^Baseline values presented. Values obtained at screening may vary.

^b^*JAK2* and *CALR*, *n* = 1; *JAK2* and *MPL*, *n* = 1; *CALR* and *MPL*, *n* = 1.

^c^Triple negative status was defined as lack of positive result for JAK2, CALR, or MPL mutation.

^d^Constitutional symptoms included weight loss, fever, and night sweats.

^e^Patient included in study based on screening values.

^f^Defined according to IWG-MRT criteria as 6 or more transfusions in the 12 weeks prior to baseline [27].

A total of 28 (54.9%) patients completed the study and 1 patient was considered as treatment ongoing at the time of data cut-off. Reasons for discontinuation of treatment included: patient decision (13.7%; *n* = 7, including: personal reasons, *n* = 2; hematopoietic stem cell transplantation, *n* = 2; withdrawal of consent, *n* = 2; and difficulties in adhering to protocol-required visit schedule, *n* = 1), physician decision (5.9%; *n* = 3, including hematopoietic stem cell transplantation, *n* = 2, and no response to treatment, *n* = 1), AEs (7.8%; *n* = 4), death (7.8%; *n* = 4), progressive disease (3.9%; *n* = 2), or protocol deviation (3.9%; *n* = 2).

### Dosing

**Fig. 2:**
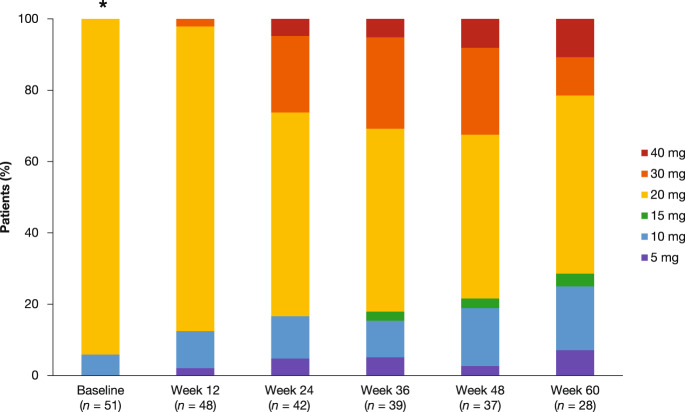
Total daily dose of ruxolitinib over time. Total daily doses were achieved as follows: 5 mg qd; 5 mg b.i.d.; 5 mg qd and 10 mg qd; 10 mg b.i.d.; 15 mg b.i.d.; 20 mg b.i.d. b.i.d twice daily; *qd* once daily. ^*^Three patients started the study at a 10 mg qd. Two of these were dosing errors that were corrected within 5 and 6 days. The third was a physician decision for a patient who did not continue with the next phase of the study due to progressive disease and was not included in subsequent analyses.

During the study, the median daily dose of ruxolitinib was 20 (range 8–36) mg and median exposure was 62.6 (range 3–92) weeks. By week 24, 26.2% (11/42 patients) received a total daily dose of ≥30 mg, and by week 48, 12 of the 37 patients with dose data (32.4%) received daily doses of 30 mg or more (Fig. 2). At final data cut-off, 12.0% (6/50) of patients had received dose increases and 30.0% (15/50) had maintained their starting dose (Supplementary Table S2). The majority of patients who maintained the starting dose achieved ≥50% reduction in spleen length at week 12 or later (11/15 patients) and 2 patients discontinued treatment prior to the first protocol mandated dose increase at week 12.

### Efficacy

**Fig. 3:**
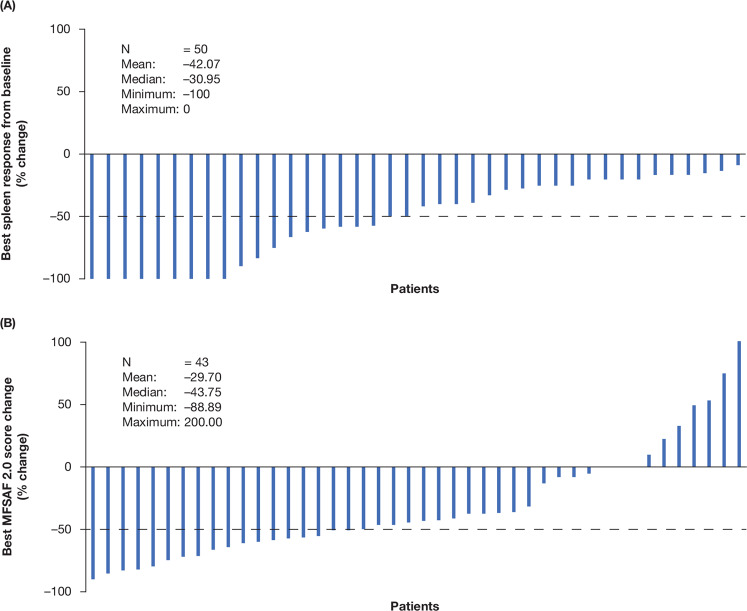
Spleen and symptom response. Best response according to spleen length (**A**) and MFSAF score change (**B**) from baseline for individual patients. Note: patient 44 achieved a best MFSAF score of +200.7%. MFSAF Myelofibrosis Symptom Assessment Form.

A total of 56% (95% CI 41.3–70.0; *n*/*N* = 28/50) of patients had a ≥50% reduction in SL by week 24. Six of the 9 (66.7%) patients who were TD at baseline met the primary endpoint of SL reduction of ≥50%, and 21 of the 40 (52.5%) baseline non-TD patients met this endpoint. When stratified by DIPSS status, 55.6% (5/9) of intermediate-1 risk, 57.1% (16/28) of intermediate-2 risk, and 40% (4/10) of high-risk patients achieved a ≥50% reduction in SL by week 24. A total of 70% (35/50) of patients achieved a ≥50% reduction in SL at any time during the study (Fig. 3A).

The baseline characteristics of patients who had a dose increase during the study were generally similar to those who did not have a dose increase (Table 2). However, a greater proportion of patients who received a dose increase had greater palpable spleen length (median 14 cm vs 9 cm) and higher median white blood cell count counts (14 × 10^9^/l vs 8.7 × 10^9^/l) than patients who did not receive a dose increase. In addition, the patients who did not receive a dose increase had a higher prevalence of low platelet counts (27.8% vs 6.7%). Of the 15 patients who received a dose increase at 12 weeks, 7 (47%) had a ≥50% reduction in spleen size at week 24 and were spleen responders.

**Table 2:** Baseline characteristics of patients who did or did not receive a ruxolitinib dose increase during the study^a^.

Characteristics	Patients with a dose increase (*n* = 15)	Patients without a dose increase (*n* = 36)
Type of MF, *n* (%)		
PMF	10 (66.7)	24 (66.7)
PPV-MF	2 (13.3)	4 (11.1)
PET-MF	3 (20)	8 (22.2)
Mutational status, *n* (%)		
MPN driver mutations^b^	14 (93.3)	30 (83.3)
Triple negative^c^	1 (6.7)	6 (16.7)
Time since initial diagnosis, median (range), months	14.6 (0.3–222)	14.9 (0.3–154.9)
Constitutional symptoms, *n* (%)	10 (66.7)	19 (52.8)
Palpable SL, median (range), cm	14 (5–35)	9 (5–18)
DIPSS category, *n* (%)		
Intermediate-1	4 (26.7)	5 (13.9)
Intermediate-2	6 (40)	22 (61.1)
High	3 (20)	7 (19.4)
Unknown	2 (13.3)	2 (5.6)
Hb level, median (range), g/dl	9.0 (7.9–11.5^b^)	8.8 (6.6–10.3^d^)
<8 g/dl, *n* (%)	1 (6.7)	8 (22.2)
≥8 g/dl, *n* (%)	14 (93.3)	28 (77.8)
Platelet count, median (range), ×10^9^/l	193 (55–657)	171 (56–762)
<100 × 10^9^/l, *n* (%)	1 (6.7)	10 (27.8)
≥100 × 10^9^/l to <200 × 10^9^/l, *n* (%)	7 (46.7)	13 (36.1)
≥200 × 10^9^/l, *n* (%)	7 (46.7)	13 (36.1)
WBC, median (range), ×10^9^/l	14 (4.5–66.3)	8.7 (2.7–71)
Blood blast cell percentage, *n* (%)		
<1%	7 (46.7)	18 (50)
≥1%	6 (40)	16 (44.4)
Missing	2 (13.3)	2 (5.6)
TD^e^	2 (13.3)	7 (19.4)

*ANC* absolute neutrophil count*; CALR* calreticulin; *DIPSS* Dynamic International Prognostic Scoring System; *Hb* hemoglobin; *IWG-MRT* International Working Group for Myeloproliferative Neoplasms Research and Treatment; *JAK2* Janus kinase 2; *MF* myelofibrosis; *MPL* myeloproliferative leukemia protein; *MPN* myeloproliferative neoplasms; *PET* post-essential thrombocythemia; *PMF* primary myelofibrosis; *PPV* post-polycythemia vera; *SL* spleen length; *TD* transfusion-dependent; *WBC*, white blood count.

^a^Protocol guidelines for dose up-titrations were based on efficacy and platelet counts provided that ANC was >500 μl and Hb level was ≥6.5 g/dl.

^b^MPN driver mutations: *JAK2*, *CALR*, and *MPL.*

^c^Triple negative status was defined as lack of positive result for *JAK2*, *CALR*, or *MPL* mutation

^d^Patient included in study based on screening values.

^e^Defined according to IWG-MRT criteria as 6 or more transfusions in the 12 weeks prior to baseline [27].

### Patient-reported outcomes

At week 24, the median percentage change in MF-7 Total Symptom Score (TSS) from baseline was −54.5% (range: −100.0% to +150.0%), with a median absolute change of 8 points, and by week 48, the median percentage change from baseline was −42.3 (range: −100.0% to +100.0%), with a median absolute change of 4 points (Supplementary Fig. S1). Scores are measured from 0 (absent) to 10 (worst imaginable), so decreased scores represent improvement in symptoms. When assessed by risk group, 75.0% (6/8), 84.6% (22/26), and 75.0% (6/8) of the intermediate-1 risk, intermediate-2 risk, and high-risk DIPSS groups, respectively, reported a ≥50% reduction in MF-7 TSS (symptom improvement) at any point during the study. Similar changes were seen in the modified MFSAF version 2.0 TSS, with the greatest median change from baseline at any time during the study of −43.8% (range: −88.9% to +200.0%) (Fig. 3B). At week 24, the median percentage change in MFSAF score from baseline was –55.2% (range: −100.0% to +145.5%), with a median absolute change of 9 points, and by week 48, the median percentage change from baseline was −45.5 (range: −100.0% to +100.0%), with a median absolute change of 7.5 points (Supplementary Fig. S2). For MFSAF scores according to risk group, 75.0% (6/8), 84.6% (22/26), and 50.0% (4/8), of the intermediate-1 risk, intermediate-2, and high-risk DIPSS groups, respectively, reported a ≥50% reduction in score at any point during the study. Using the PGIC score, a total of 82.9% (34/41) of patients reported an improvement at week 24 and 87.9% (29/33) at week 48 (Supplementary Fig. S3).

### Safety

Overall, at least 1 AE of any grade was reported in 44/51 subjects (86.3%) and 26/51 (51.0%) were considered treatment-related. Five patients experienced AEs leading to treatment discontinuation: anemia worsening (grade 3); leukocytosis (grade 3); Crohn’s disease reactivation (grade 4); bacterial lower respiratory tract infection (grade 5); and sepsis (grade 4). In total, 25 (49.0%) patients had at least 1 dose reduction and 16 (31.4%) had at least 1 dose interruption (Fig. 2). AEs were the most common reason for both dose reductions and interruptions. The most frequent AEs leading to dose interruption/adjustment were thrombocytopenia (17.6% [9/51] of patients) and anemia (11.8% [6/51] of patients), which were also the most frequent AEs occurring across the study population (Table 3). During the study period, 1 patient experienced a thrombotic event (arterial retinal thrombosis) and another had progression to acute myeloid leukemia.

**Table 3:** Adverse events occurring in ≥5% of patients by MedDRA preferred term.

MedDRA preferred term	All grades, *n* (%)	Grade ≥3, *n* (%)
Anemia	18 (35.3)	16 (31.4)
Thrombocytopenia	15 (29.4)	10 (19.6)
γ-glutamyltransferase increase	6 (11.8)	2 (3.9)
Asthenia	6 (11.8)	1 (2.0)
Diarrhea	6 (11.8)	0 (0.0)
ALT/AST increase	5 (9.8)	0 (0.0)
Fatigue	5 (9.8)	0 (0.0)
Urinary tract infection	5 (9.8)	0 (0.0)

*ALT* alanine aminotransferase; *AST* aspartate aminotransferase; *MedDRA* Medical Dictionary for Regulatory Activities.

In total, 8 patients died, with 7 of those deaths occurring during the study or the study safety follow-up period. Primary reasons for death were: infections and infestations (*n* = 4), cardiac failure (*n* = 1), multiple organ dysfunction syndrome in the context of sepsis due to *Escherichia coli* (*n* = 1), and progression of MF to acute myeloid leukemia (*n* = 1). Of these, 4 patients had discontinued treatment due to AEs. No new AEs were observed in the REALISE trial compared with previous trials of ruxolitinib in MF [23, 24].

### Changes in hemoglobin levels and platelet counts

**Fig. 4:**
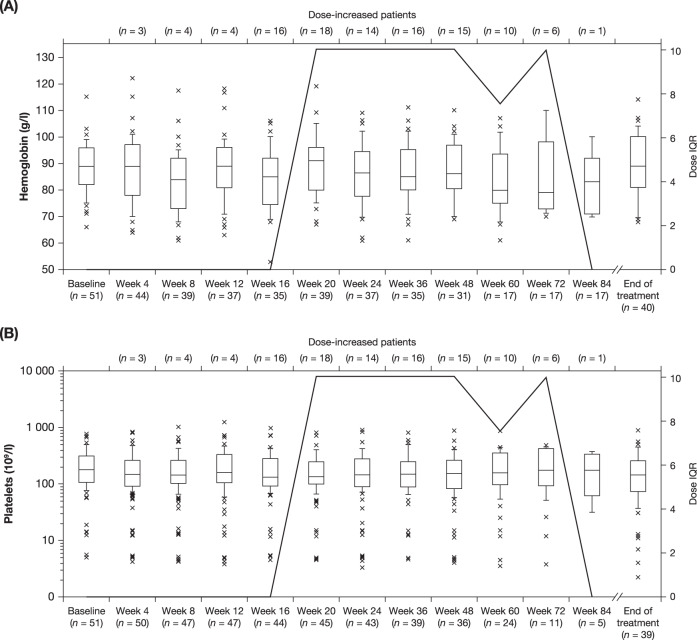
Evolution of hemoglobin and platelets. Median hemoglobin (**A**) and platelet (**B**) levels over time. Boxes indicate 25th–75th percentiles and median daily dose is indicated as a horizontal line. Whiskers indicate 10th–90th percentiles. Values outside this range are not displayed. X marks indicate values 1.5x the IQR above Q3 and 3x the IQR below Q1. Continuous line indicates IQR of change in total daily dose from starting dose. IQR interquartile range, Q1 first quartile; Q3 3rd quartile.

Hb decreases were observed in 82.4% (42/51) of patients, 54.9% (28/51) of which were grade 3. Of the patients with a grade 3 Hb decrease, 1 patient entered the trial with a grade 1 decreased Hb count and 23 and 4 patients entered the trial with a grade 2 or grade 3 decreased Hb counts, respectively. Median Hb levels remained stable throughout the study, with support of red blood cell (RBC) transfusions as needed (Fig. 4A). Platelet decreases were seen in 66.7% (34/51) of patients (17.6% [9/51] grade 3 or 4). Of the 6 patients with a grade 3 platelet decrease, 4 and 2 entered the trial with a grade 1 or grade 2 decreased platelet counts respectively, and of the 3 patients with a grade 4 platelet decrease, 2 and 1 entered the trial with a grade 1 or grade 2 decreased platelet count, respectively. A total of 11.8% (6/51) of patients required platelet transfusions during the study. After an initial drop post-baseline, median platelet counts remained stable throughout the study among those patients who remained on study (Fig. 4B). Platelet counts and hemoglobin levels were similar between patients who received a dose increase and those who did not receive a dose increase over the course of the study (Supplementary Fig. S4).

### Transfusion requirements

**Fig. 5:**
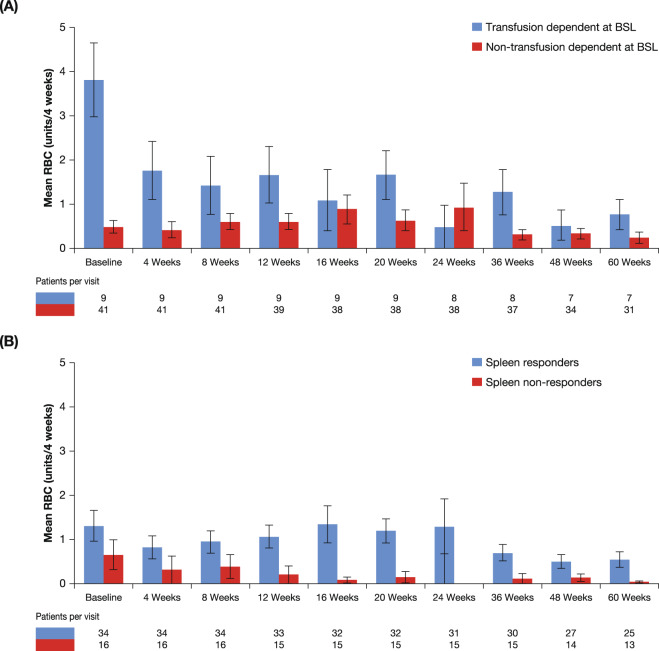
Transfusion requirements. Mean number of RBC units received during the study in patients who were transfusion-dependent or transfusion-independent at baseline (**A**) and spleen responders and non-responders at any time during the study (**B**). BSL baseline spleen length; RBC red blood cells.

During the study 66.7% (34/51) of patients received all transfusion components (whole blood transfusions, packed RBC transfusions and platelet transfusions). Of the 8 patients who received erythropoietin during the study, 6 started prior to the study; 1 patient received erythropoietin prior to the study, but discontinued after study commencement. Additionally, based on the physician’s decision, 11 patients received low-dose corticosteroids during the study period, usually as a single dose during surgery or as premedication. The mean number of RBC units received in the prior 4 weeks was highest at baseline in TD patients, at approximately 3.8 units, which decreased to between 0.5 and 1.8 units until around week 48, before further decreasing to <1 unit/4 weeks prior to subsequent visits (Fig. 5A). Mean RBC units were lower in non-TD patients in the 4 weeks prior to baseline at approximately 0.5 units, and remained between 0.5 and 1.0 units until week 36, after which the mean dropped to <0.3 units/4 weeks (Fig. 5A). As shown in Fig. 5A, overall, the requirements for RBC transfusions decreased for TD patients and remained at similar levels throughout the study for non-TD patients. There was a trend toward decreasing transfusion requirement both in patients who did and those who did not achieve a spleen response (Fig. 5B). Patients who were TD at baseline, but did not have a spleen response, showed a trend toward decreasing RBC transfusion requirement (Supplementary Fig. S5A). RBC transfusions according to dose increase are shown in Supplementary Fig. S5B.

## Discussion

Anemia is a frequent manifestation of MF. Its current treatments include androgens, erythropoiesis stimulating agents (ESA), immunomodulatory agents such as thalidomide and lenalidomide, and prednisone [9], but they are not effective in many patients, especially in those with transfusion dependence. Therefore, new therapies for the anemia of patients with MF are needed. Luspatercept is an agent relieving blockade of the terminal stages of erythropoiesis that has shown efficacy in the anemia of low-risk myelodysplastic syndromes, and has shown promising results in a phase 2 clinical trial in anemic patients with MF [20] and is moving into a phase 3 study in combination with ruxolitinib. Moreover, in a recent phase 2 trial, the JAK1/JAK2 inhibitor momelotinib has shown efficacy in the anemia of MF [21] and a phase 3 trial comparing this drug with danazol is currently in progress.

Therapy with ruxolitinib, a potent JAK1/JAK2 inhibitor approved for the treatment of MF-related splenomegaly or symptoms, can be associated with dose-dependent anemia, mostly emerging in the first 12 weeks of treatment. It must be remarked that patients on ruxolitinib often tolerate even very low Hb levels, which might be related to the fact that increased inflammatory cytokine levels contribute to a poorer tolerability of the anemia. However, overall, anemia under ruxolitinib therapy poses a clinical problem for a proportion of patients with MF. Clinical experience with ruxolitinib in MF has led to the suggestion that a lower starting dose of 10 mg b.i.d. with up-titration according to blood parameters may reduce the impact of treatment-related anemia while maintaining therapeutic response [18, 19]. In this context, the REALISE study was designed to evaluate a novel dosing strategy of ruxolitinib in MF patients with significant anemia. The regimen consisted of a reduced initial ruxolitinib dose with assessment of response at 12 weeks in order to apply a dose escalation if the response was not satisfactory and platelet counts remained over 100 × 10^9^/l. Overall, a 10 mg b.i.d. starting dose with up-titration after 12 weeks of treatment, if necessary and possible, was efficacious and well tolerated in anemic patients with MF, including TD patients. Of note, almost half of the patients who received a dose increase at 12 weeks of treatment because of insufficient response subsequently achieved a spleen response. A trend toward higher response rates in patients receiving titrated doses ≥10 mg b.i.d. was first highlighted in the ruxolitinib phase 1–2 trial [29]. While this study confirmed a dose–response relationship, the efficacy of a 10 mg b.i.d. dose in reducing MF-associated splenomegaly and symptoms has also been observed in independent studies [30]. In addition, with this novel dosing strategy, the majority of patients in the study experienced a significant reduction in splenomegaly and improvements in MF-associated symptoms and patient-reported outcomes, despite the presence of anemia at baseline. These results are comparable to those reported in the phase 3 studies of ruxolitinib [23, 24, 31, 32] and in an analysis of data pooled from the COMFORT trials [25]. These studies suggested that both patients who were anemic at baseline and those who became anemic during ruxolitinib treatment had a survival advantage compared with those patients receiving the comparator, best available therapy.

With the alternative dosing strategy used in the REALISE study, spleen response was seen at week 24 in anemic patients, regardless of transfusion dependence. The number of RBC transfusions decreased in TD patients, but remained relatively consistent in non-TD patients. Hb levels remained reasonably stable throughout the study, with the support of RBC transfusions as needed. As previously mentioned, it has been seen in clinical trials [23, 24, 31, 32] that during the first months of ruxolitinib treatment patients with MF often show an accentuation of the anemia, while later on Hb levels tend to slightly improve or to return to the baseline values. This may partly reflect dose reductions or early discontinuation of high-risk patients. Reduced transfusion burden may be attributed to an improvement in overall performance status related to the inhibition of the cytokines. The reduction of splenomegaly that follows ruxolitinib treatment might also contribute to the improvement in anemia. Additionally, starting with a lower ruxolitinib dose may have altered the rate of the Hb decline and nadir by decreasing the levels of JAK-mediated inhibition of hematopoiesis. Concerning other side effects, ruxolitinib was well tolerated and no new safety signals were identified.

Some patients in this study received ESAs during ruxolitinib therapy. In a post hoc analysis of the COMFORT-2 study, of the 13 patients who received ESA therapy during the study by decision of their treating physician, 7 showed an improvement in their Hb values and 2 of the 6 who were transfusion dependent at the start of ESA therapy had a decrease in the number of packed RBC transfusions needed, but without becoming transfusion independent [33]. Therefore, the possible effect of ESA therapy on the evolution of the anemia in this subgroup of patients of the present study is difficult to determine.

Two baseline characteristics of this study, time from diagnosis to initiation or ruxolitinib treatment (median 14.9 months) and patients receiving alternative treatment prior to ruxolitinib (28/51 patients), could indicate a trend toward delaying ruxolitinib treatment initiation, or that it is not considered first therapy of choice in patients for whom it may be indicated. Of the 28 patients who received prior treatment, 21 had received hydroxyurea prior to ruxolitinib. In this context, rapid initiation of ruxolitinib treatment is supported by evidence from a subgroup analysis of the JUMP trial (38% of whom were anemic) in which better spleen response was predicted for patients who received ruxolitinib as first-Iine treatment [34].

Based on these results, we propose that this new dosing strategy may be appropriate for those MF patients commencing ruxolitinib therapy due to splenomegaly and/or MF-associated symptoms who have clinically relevant anemia (Hb < 10 g/dl). Data from multiple studies have demonstrated the benefit of ruxolitinib in MF patients, regardless of baseline hemoglobin levels, and this study shows that alternative dosing strategies can be considered in order to avoid delaying the initiation of ruxolitinib treatment in anemic MF patients with symptoms who can derive benefit from immediate initiation of treatment [23, 24, 31–33, 35]. One important clinical question that could not be addressed by this study is whether this dose-titration strategy reduced the severe thrombocytopenia and treatment-emergent anemia observed in previous randomized clinical trials [23, 24]. Unfortunately, due to the fact that the present trial was non-randomized and included only anemic patients at baseline, the results of the above studies are not directly comparable, since a cohort of exclusively anemic patients may be at higher risk of cytopenia than the population of the COMFORT studies, which also included patients who were not anemic.

It is also noteworthy that patients requiring a ruxolitinib dose increase tended to have larger spleens than those who did not. Thus, in anemic MF patients who need JAK inhibitor therapy, the choice of the initial ruxolitinib dose (i.e., the standard dose, as dictated by platelet counts, or a lower dose with escalation at 12 weeks, if needed) could be based on the degree of need to achieve a rapid spleen reduction. Therefore, in case of moderate-to-marked, but not massive, splenomegaly, the possibility of adopting a 2-step dosing policy could be considered. Finally, in anemic patients with constitutional symptoms, but without significant splenomegaly, selection of a lower starting dose of ruxolitinib (i.e., 10 mg b.i.d.) is a reasonable and feasible strategy.

In conclusion, the results of the REALISE study demonstrate that an alternative dosing regimen of ruxolitinib in anemic MF patients is effective and well-tolerated, reinforcing the notion that, in MF patients with splenomegaly and/or constitutional symptoms, it is not necessary to delay or withhold treatment with ruxolitinib because of co-existent or treatment-emergent anemia.

## Supplementary information

Supplementary Materials
