# Supplementary material for: Efficacy and safety of a novel dosing strategy for ruxolitinib in the treatment of patients with myelofibrosis and anemia: the REALISE phase 2 study
Source: Leukemia. 2021 May 20;35(12):3455–65. doi: 10.1038/s41375-021-01261-x (PMC8632662; doi:10.1038/s41375-021-01261-x)
Supplement: Supplementary file 1 — Supplementary Materials [file 41375_2021_1261_MOESM1_ESM.docx]

**Efficacy and safety of a novel dosing strategy for ruxolitinib in the treatment of patients with myelofibrosis and anemia: The REALISE phase 2 study**

Francisco Cervantes, David M. Ross, Atanas Radinoff, Francesca Palandri, Alexandr Myasnikov, Alessandro M. Vannucchi, Pierre Zachee, Heinz Gisslinger, Norio Komatsu, Lynda Foltz, Francesco Mannelli, Francesco Passamonti, Geralyn Gilotti, Islam Sadek, Ranjan Tiwari, Evren Zor, Haifa Kathrin Al-Ali

**Supplementary Tables**

**Table S1**. Additional baseline patient characteristics

| Characteristics | All patients (*N* = 51) |
| --- | --- |
| BMI, median (range), kg/m^2^ | 24.5 (18.0–35.0) |
| Bone marrow fibrosis grade at diagnosis, n (%)^a^ |  |
| 1 | 6 (11.8) |
| 2 | 26 (51.0) |
| 3 | 18 (35.3) |
| Missing | 1 (2.0) |
| Previous transfusion, n (%) | 23 (45.1) |
| Erythrocyte mean corpuscular Hb concentration, median (range), mmol/l | 4.9 (0.5–19.4) |
| Erythrocyte mean corpuscular volume, median (range),fl | 88.4 (62.0–110.4) |
| Erythrocytes, median (range), × 10^12^/l | 3.0 (2.2–4.6) |
| Hematocrit, median (range) | 0.3 (0.2–0.4) |
| Leukocytes, median (range), × 10^9^/l | 9.9 (2.7–71.0) |
| Neutrophils, median (range), × 10^9^/l | 7.0 (1.3–58.0) |
| Blast cell %, median (range) | 0 (0–9.0), *n = 47* |
| Monocytes, median (range), × 10^9^/l | 0.4 (0–5.4) |
| Alanine aminotransferase, median (range), U/l | 16.0 (3.0–36.0) |
| Aspartate aminotransferase, median (range), U/l | 20.0 (10.0–44.0) |
| Alkaline phosphatase, median (range), U/l | 97.0 (30.0–448.0) |
| Bilirubin, median (range), umol/l | 14.7 (3.4–74.9) |
| Lactate dehydrogenase, median (range), U/l | 663.0 (139.0–2194.0) |
| Albumin, median (range), g/l | 42.0 (27.9–56.1) |
| Potassium, median (range), mmol/l | 4.4 (3.6–6.1) |
| Sodium, median (range), mmol/l | 140.2 (129.0–147.0), *n = 50* |

^a^Graded by the European Consensus grading system (Thiele J, et al, *Haematologica* 2005;90:1128–1132).

*BMI* body mass index

**Table S2.** Adverse events leading to dose interruption or adjustment

| Patient | SAE? | Preferred term | Start day | Duration (days) | Grade | Related? | Action | Outcome |
| --- | --- | --- | --- | --- | --- | --- | --- | --- |
| 1 | N | Thrombocytopenia | 91 | 14 | 3 | Y | Dose reduction | Resolved |
| 2 | N | Thrombocytopenia | 259 | 10 | 2 | Y | Dose reduction | Recovering |
|  | N | Thrombocytopenia | 269 | - | 1 | Y | Dose increased | Not resolved |
|  | N | Retinal vascular disorder | 369 | 25 | 2 | Y | Dose reduction | Resolved |
| 3 | N | Anemia | 124 | - | 3 | N | Dose reduction | Resolved |
|  | Y | Bronchitis | 191 | - | 3 | N | Drug interruption | Not resolved |
|  | N | Thrombocytopenia | 195 | 64 | 4 | Y | Drug interruption | Resolving |
|  | Y | Anemia | 271 | 2 | 4 | Y | Drug interruption | Resolved |
|  | N | Anemia | 273 | 2 | 3 | Y | Drug interruption | Resolving |
| 4 | N | Thrombocytopenia | 69 | 16 | 3 | Y | Dose reduction | Resolving |
| 5 | N | Pyrexia | 299 | 15 | 2 | N | Drug interruption | Recovered |
|  | Y | Pneumonia | 389 | 14 | 3 | N | Drug interruption | Not resolved |
| 6 | N | Thrombocytopenia | 166 | 29 | 2 | Y | Dose reduction | Resolving |
| 7 | N | Thrombocytopenia | 23 | 156 | 3 | Y | Dose reduction | Not resolved |
|  | Y | Bile duct stone | 106 | 29 | 3 | N | Drug interruption | Resolved |
|  | N | Thrombocytopenia | 179 | 26 | 4 | Y | Drug interruption | Resolving |
|  | N | Thrombocytopenia | 424 | 14 | 3 | N | Dose reduction | Resolving |
| 8 | Y | Aortic valve disease | 136 | 28 | 4 | N | Drug interruption | Resolved |
|  | N | Pruritus | 337 | 120 | 2 | N | Dose increased | Resolved |
|  | N | Thrombocytopenia | 453 | 11 | 3 | Y | Dose reduction | Resolved |
|  | Y | Anemia | 575 | 5 | 3 | N | Drug interruption | Resolved |
| 9 | N | Neutropenia | 250 | 3 | 4 | N | Drug interruption | Resolved |
| 10 | N | Thrombocytopenia | 508 | - | 2 | Y | Dose reduction | Not resolved |
| 11 | N | Thrombocytopenia | 334 | 83 | 2 | Y | Dose reduction | Resolved |
| 12 | Y | Urinary tract infection | 64 | 12 | 3 | Y | Dose reduction | Resolved |
| 13 | N | Nasal herpes | 127 | 15 | 2 | Y | Dose reduction | Resolved |
|  | N | Thrombocytopenia | 337 | 84 | 3 | Y | Dose reduction | Unknown |
| 14 | Y | Lower respiratory tract infection | 193 | 3 | 3 | N | Drug interruption | Not resolved |
| 15 | N | Thrombocytopenia | 61 | - | 2 | Y | Dose reduction | Resolving |
|  | N | Anemia | 79 | 90 | 2 | Y | Dose reduction | Resolved |
| 16 | Y | Hepatic encephalopathy | 143 | 10 | 3 | N | Drug interruption | Resolved |
|  | Y | Hepatic encephalopathy | 251 | 6 | 4 | N | Drug interruption | Unknown |
| 17 | N | Thrombocytopenia | 303 | 38 | 2 | Y | Dose reduction | Resolved |
| 18 | N | Anemia | 337 |  | 3 | Y | Dose reduction | Not resolved |
|  | N | Herpes zoster | 421 |  | 2 | Y | Dose reduction | Not resolved |
| 19 | N | Anemia | 29 | 85 | 3 | Y | Dose reduction | Resolved |
|  | N | Anemia | 148 | 63 | 3 | Y | Dose reduction | Resolved |
|  | N | Anemia | 246 | 63 | 3 | Y | Dose reduction | Resolved |
| 20 | N | Anemia | 64 | 112 | 3 | Y | Dose reduction | Resolved |
| 21 | N | Thrombocytopenia | 84 | 16 | 3 | Y | Dose reduction | Resolved |

*SAE* serious adverse event

**Supplementary figures**


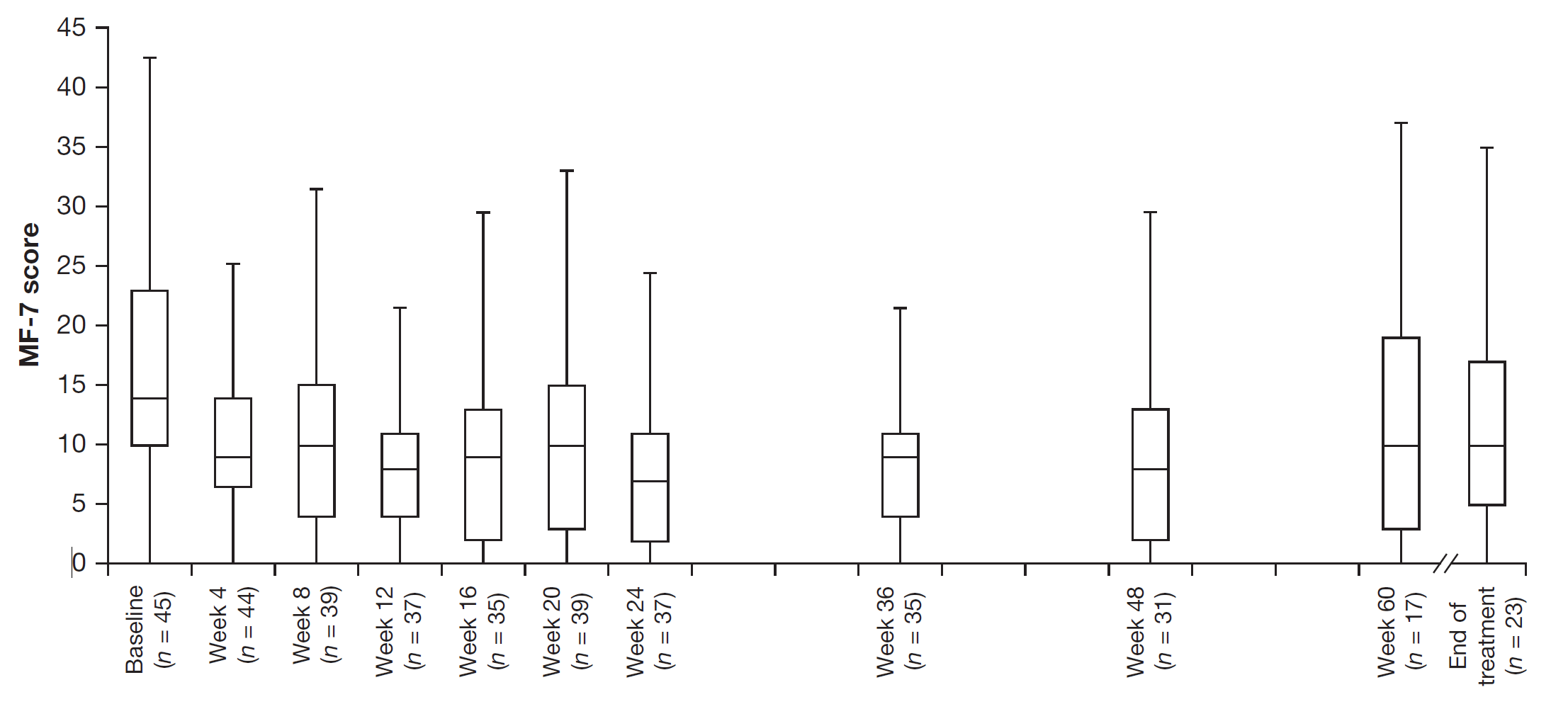


**Fig. S1. MF-7 total symptom score response over time.** MF-7, 7-point myelofibrosis score.


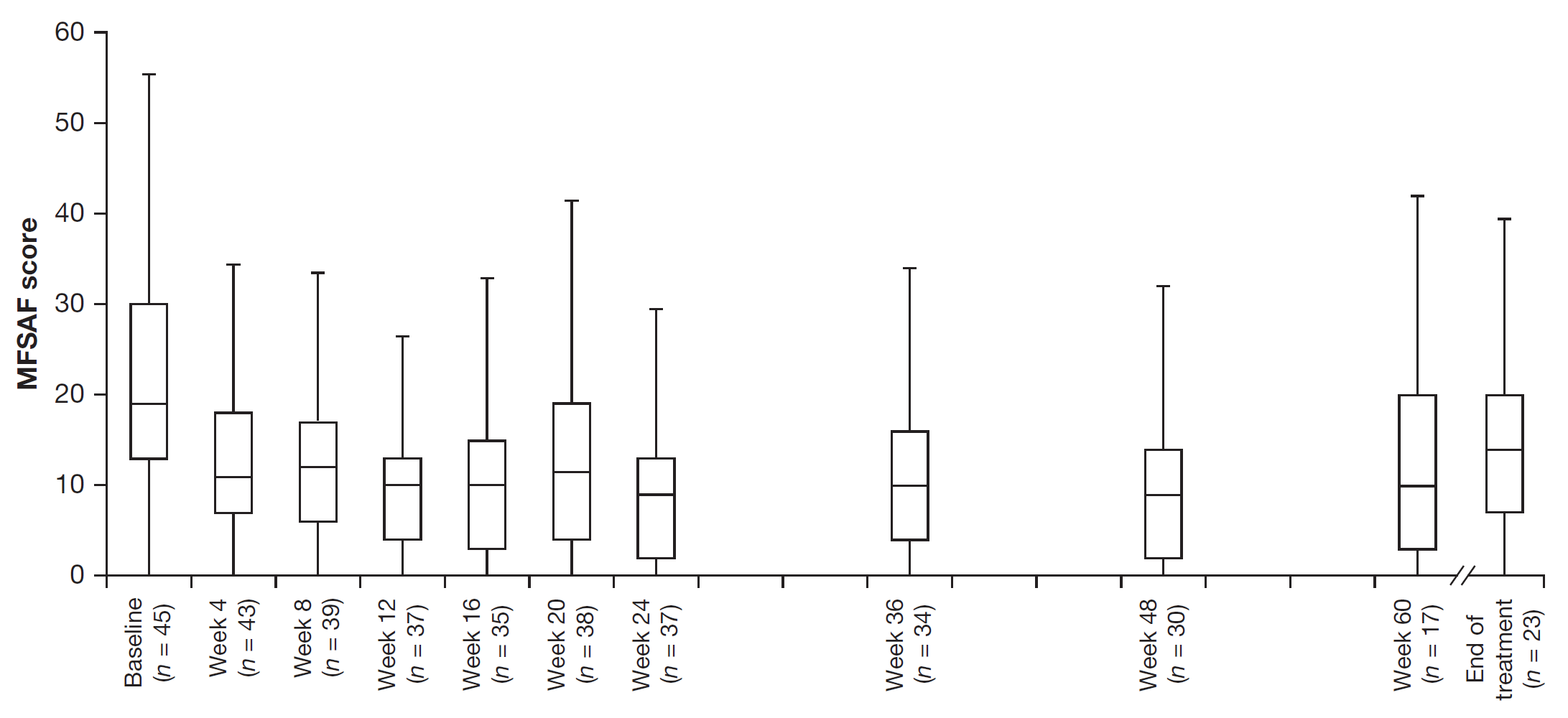


**Fig. S2. Modified MFSAF v2.0 total symptom score response over time.** MFSAF, Myelofibrosis Symptom Assessment Form.


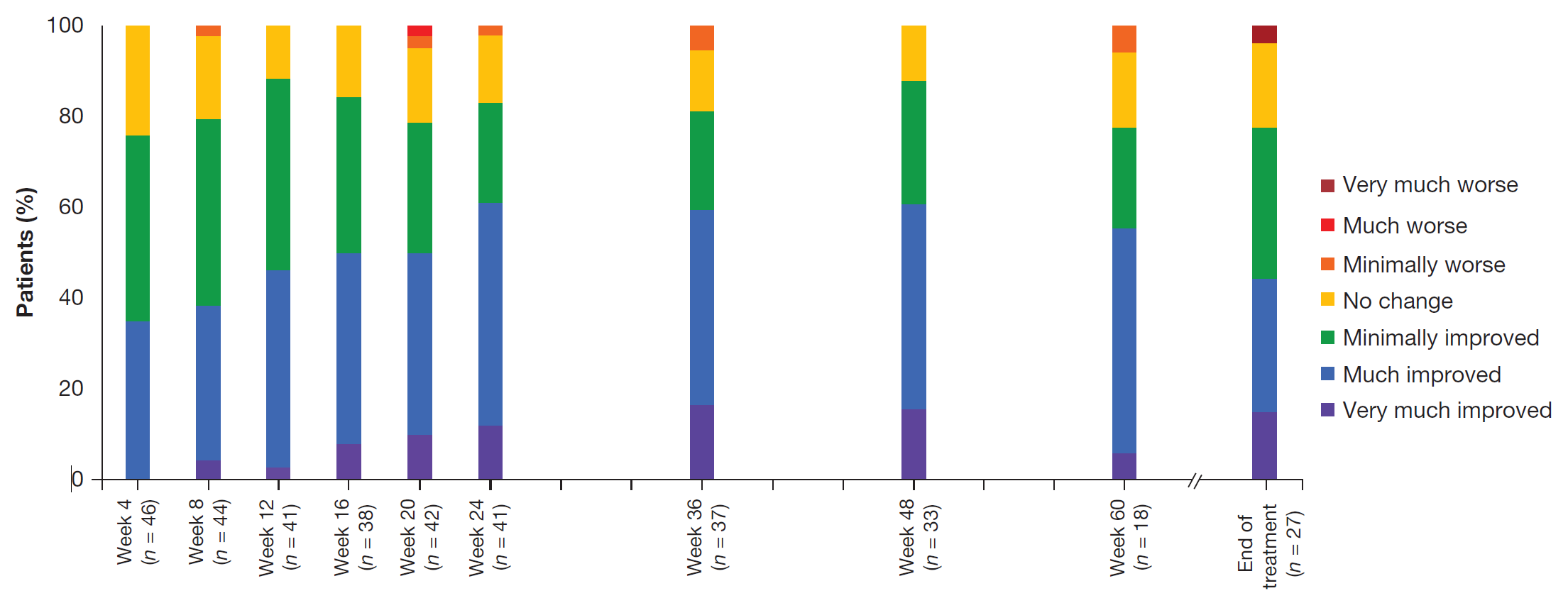


**Fig. S3. PGIC response over time.** PGIC, Patient Global Impression of Change.


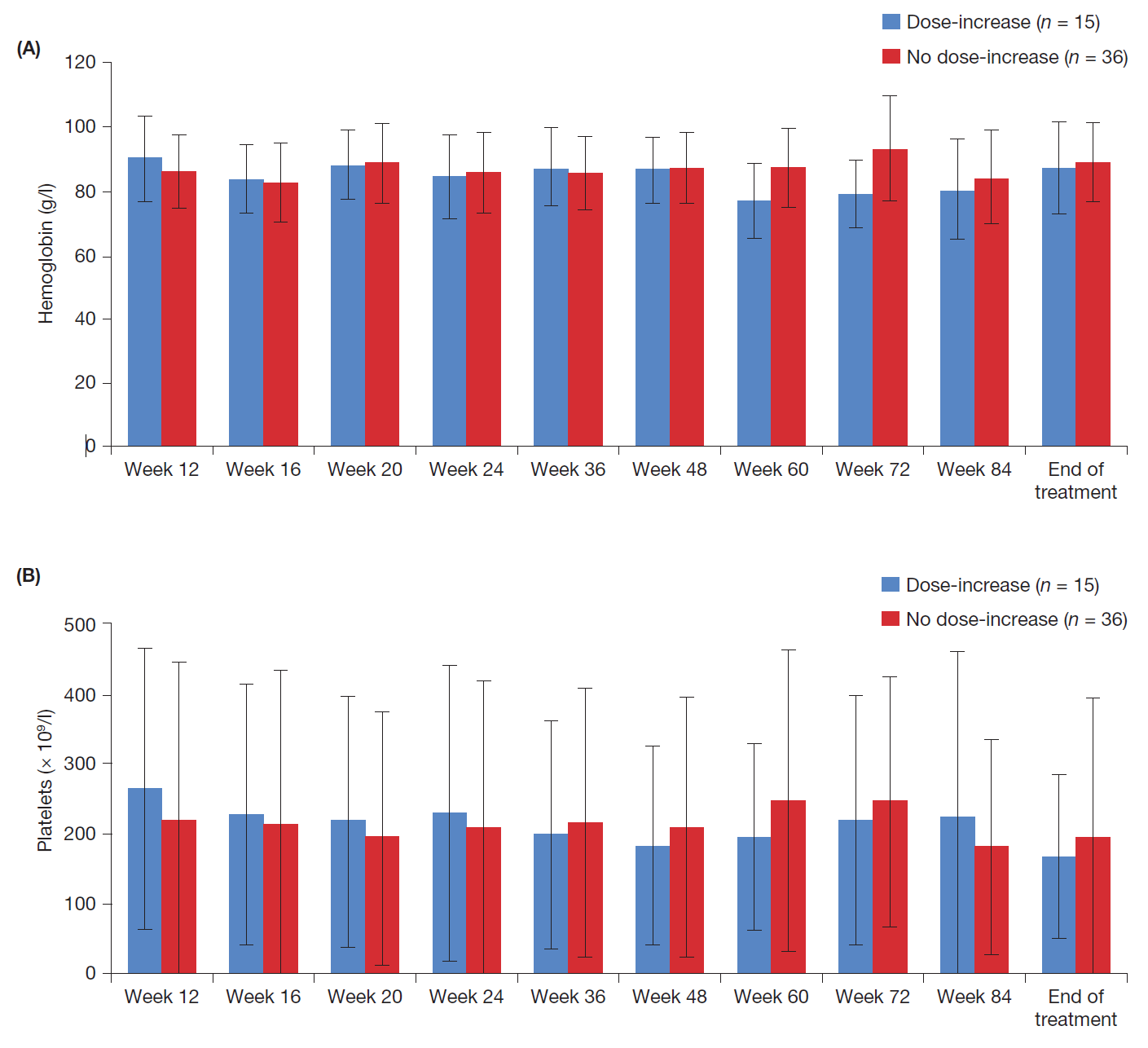


**Fig. S4. Hematological parameters over time.** (A) Hemoglobin levels. (B) Platelet levels.


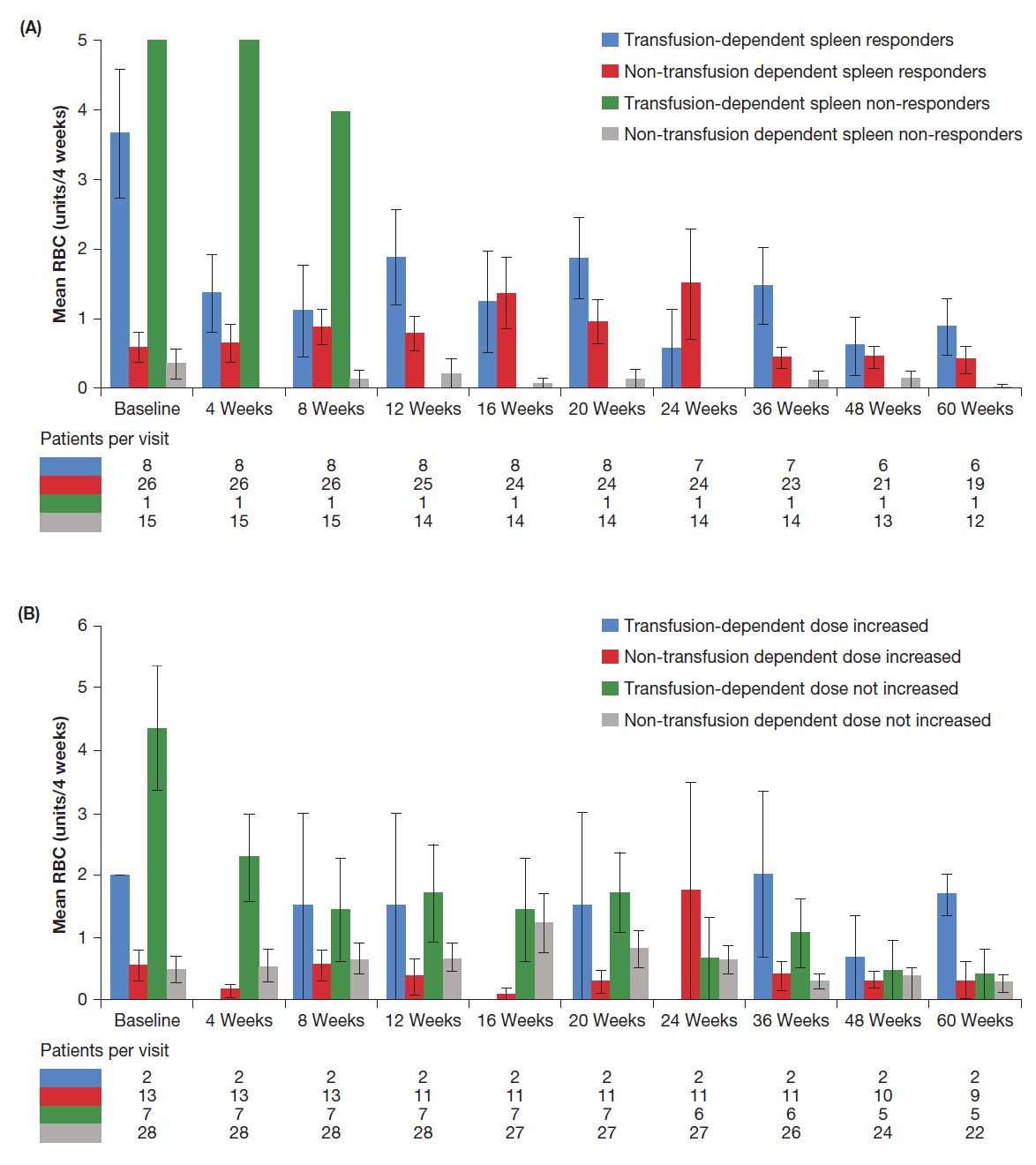


**Fig. S5. Mean number of RBC units received during the study in patients who were transfusion dependent (blue) or transfusion independent (red) at baseline according to spleen response and dose increase. (**A) Transfusion dependence and spleen response at any time. (B) Transfusion dependence and dose increase. RBC, red blood cells.
